# Bathing

**Published:** 1886-12-18

**Authors:** 


### Till the Doctor Comes.

[Inquiries and Communications for this column should be addressed, "Physician," care of the Editor of The Hospital.]

### VI.—BATHING AND BEDS.

This must only be done under instructions from the doctor. Get everything ready before disturbing the patient, as quickly and noiselessly as possible, and avoid all undue exposure, especially during the operation of drying. The following list will be useful :—

| | |
|---|---|
| Cold bath . . | 40° to 60° Fahr. |
| Tepid bath . . | 70° „ 80° „ |
| Warm bath . . | 80° „ 95° „ |
| Hot bath . . | 96° „ 100° „ |

On no account have the temperature over 100° Fahr. unless specially ordered.

Bed-making.

Have all sheets, blankets, changes of linen, etc., well aired immediately before use. In all bad cases with much hæmorrhage or discharge, keep a clean mackintosh under such part of the patient as is requisite, and place a draw-sheet over it. The draw-sheet is one of a nurse's most serviceable agents, keeping the patient dry, and protecting the bed; in some cases it requires changing frequently, and it is of the utmost importance that this should be done without the least possible disturbance. A soft old sheet having been folded to the required width (generally two feet), let the sheet be rolled up at one end, leaving just sufficient of it to pass under the patient's buttocks. When the sheet is wet, draw it through from the side opposite to the one under which it was first passed, unrolling just enough of the clean end to secure a dry piece under the buttocks. The soiled end may then be rolled up and tightly pinned. In this way one draw-sheet will be sufficient for several changes, and by pinning a clean one to it a succession of draw-sheets may be passed under a patient with the least possible disturbance. When it is necessary to change the bedclothes of a bed-ridden or nearly helpless patient, the following will be found an easy course to pursue :—Having a clean sheet half rolled up, turn the patient on one side, roll up the dirty under sheet as close to the patient as possible, and place the unrolled half of the clean sheet over that portion of the bed from which the dirty linen has been removed; then turn your patient on to this, and having removed the remainder of the dirty sheet, and replaced it by unrolling the clean one, the patient will be made comfortable very rapidly and with the least possible inconvenience. If the patient is too weak to be thus moved, it is not difficult to change the under sheet without much disturbance, provided the aid of an assistant is secured. With this method it is necessary to begin at the head of the bed, to gradually roll up the dirty sheet, and at the same time to replace it with the clean one, which must be rolled up and put in readiness at the head of the bed before the dirty linen is removed. With a little practice this may be done quickly, and without any discomfort to the patient. In surgical cases, fractures, etc., the patient may grasp the bed-pull, and thus raise himself sufficiently to allow the sheets to be changed without any trouble or delay.

The Bed-pan. Should be covered with loose flannel and kept near the fire. The slipper shape is the one more easily used. Introduce it at the side of the bed, bend the patient's knees, and slip it under him. Place some disinfectant powder in it before use; cover it afterwards with some more powder, and remove it immediately from the room.

The Feeding-cup. Should have a nicely-curved spout, better if at right angles with the handle. Scald it after use.

